# The effect of age on the magnitude and longevity of Th1‐directed CD4 T‐cell responses to SARS‐CoV‐2

**DOI:** 10.1111/imm.13475

**Published:** 2022-04-22

**Authors:** Ryan G. Nattrass, Lisa Krafft, Polina Zjablovskaja, Marc Schuster, Bahram Kasmapour, Cem Sarisoy, Jessica Minich, Elena Bach, Hendrik Streeck

**Affiliations:** ^1^ Institute of Virology University Hospital Bonn Bonn Germany; ^2^ German Centre for Infection Research (DZIF) partner site Bonn‐Cologne Braunschweig Germany; ^3^ Miltenyi Biotec Bergisch Gladbach Germany

**Keywords:** age, CD4 T cell, flow cytometry, SARS‐CoV‐2, Th1

## Abstract

Age is associated with changes in the immune system which increase the risk for severe COVID‐19. Here, we investigate SARS‐CoV‐2‐reactive CD4 T cells from individuals recovered from SARS‐CoV‐2 infection with mild COVID‐19 symptoms after 3, 6 and 9 months using incubation with SARS‐CoV‐2 S1, S2 and N‐peptide pools, followed by flow cytometry for a Th1‐activation profile or proliferation analyses. We found that SARS‐CoV‐2‐reactive CD4 T cells are decreasing on average after 9 months but highly polyfunctional CD4 T cells can peak after 6‐month recovery. We show that individuals older than 60 years of age have significantly more SARS‐CoV‐2‐reactive T cells in their blood after 3 months of recovery compared to younger individuals and that the percentage of SARS‐CoV‐2‐reactive Th1‐directed CD4 T cells in the blood of mild‐COVID‐19‐recovered individuals correlates with age. Finally, we show that individuals over the age of 40 have significantly increased the amounts of highly polyfunctional SARS‐CoV‐2‐S‐peptide‐reactive CD4 T cells, compared to SARS‐CoV‐2 naïve individuals, than those under the age of 40. These findings suggest that in individuals recovered from mild COVID‐19, increased age is associated with significantly more highly polyfunctional SARS‐CoV‐2‐reactive CD4 T cells with a Th1‐profile and that these responses persist over time.

## Graphical abstract

A large cohort of un‐vaccinated, mild COVID‐19‐recovered individual's Th1‐directed CD4 T cell responses to SARS‐CoV‐2 peptides were analysed with regards to response magnitude and longevity alongside age. Th1‐directed CD4 T cell responses were detectable at least nine months after recovery in all age groups, however, significantly more robust poly‐functional responses were detected within older age‐groups which persisted over time.

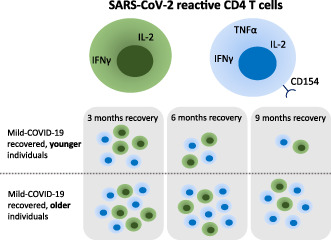

**Abbreviations**
Adv5: human adenovirus 5
BrdU: bromodeoxyuridine
CD: cluster of differentiation
CMV: cytomegalovirus
COVID‐19: coronavirus disease 19
DMSO: dimethyl sulfoxide
EBV: Epstein–Barr virus
ELISA: enzyme‐linked immunosorbent assay
h: hours
IAC: intra‐assay control
IFNγ: interferon gamma
IgA: immunoglobulin A
IgG: immunoglobulin G
IL‐2: interleukin 2
N: SARS‐CoV‐2 “N”‐peptide pool
PBMC: peripheral blood mononuclear cell
PCR: polymerase chain reaction
PHA: phytohaemagglutinin
POD: peroxidase
RPMI: Roswell Park Memorial Institute
S1: SARS‐CoV‐2 “S1”‐peptide pool
S2: SARS‐CoV‐2 “S2”‐peptide pool
SARS‐CoV‐2: severe acute respiratory syndrome coronavirus 2
Th1: type‐1 T helper; Th2, type‐2 T helper
TNFα: tumour necrosis factor alpha
YO: years old

## INTRODUCTION

The novel human pathogen severe acute respiratory syndrome coronavirus 2 (SARS‐CoV‐2) [1] which causes coronavirus disease 19 (COVID‐19) has led to significant worldwide mortality since its discovery in late 2019.

Evidence, thus, far has indicated that the severity of COVID‐19 after SARS‐CoV‐2 infection is linked to inefficient virus‐specific immune responses. More severe COVID‐19 cases have been linked with ineffective innate immunity [2, 3] which can lead to a delayed adaptive immune response and a higher viral burden, a situation which has been associated with fatal cases of COVID‐19 [4]. Furthermore, adaptive immune response involving the presence of detectable SARS‐CoV‐2‐specific T cells in convalescent individuals has been significantly associated with milder disease [5, 6, 7]. Evidently, clearance of SARS‐CoV‐2 is dependent upon adaptive immune responses to SARS‐CoV‐2 antigens. Effective T‐cell immunity has also been associated with the absence of disease progression for multiple viral infections and, specifically, the ability of polyfunctional T cells to generate multiple cytokines and activation markers upon stimulation with viral antigen [8, 9]. Virus‐specific T cells commonly develop a Th1 profile, which have well‐documented anti‐viral properties through the secretion of cytokines such as IFNγ, TNFα and IL‐2 and which have shown to be protective against lethal SARS‐CoV infection in mice [10].

Age has been identified as one of the largest risk factors for COVID‐19 mortality with significantly increased rates of fatalities in individuals aged 65 years or older [11, 12] and mortality from SARS‐CoV‐2‐infection being approximately 10 times higher for a 40‐year old compared to a 20‐year old [13]. Ageing is associated with increased T‐cell senescence, which may contribute to inefficient responses to viral infections [14]; however, conversely, senescence is also associated with increased T‐cell inflammatory responses [15], which may add to the immunopathology of COVID‐19. Several studies have demonstrated that age is associated with a perturbed and reduced functional immune response to SARS‐CoV‐2 infection involving both the innate and adaptive immune system, which leads to increased inflammation and adverse COVID‐19 outcome [16, 17]. Furthermore, increased age is also associated with a decrease in naïve T cells [18] and COVID‐19 severity has been inversely correlated with naïve T‐cell frequency [5]. The ability of an immune system responding to a novel virus relies upon the repertoire of naïve T cells available and so an older individual is less likely to have an immune system as capable to respond as a younger one [19]. This may lead to a slower T‐cell response and more severe course of disease.

Whilst increased age is associated with an increased likelihood of severe COVID‐19, the effect of age on the adaptive immune response to SARS‐CoV‐2 in those individuals who do not develop severe symptoms is less well studied. We, therefore, investigate whether Th1‐directed CD4 T‐cell responses to SARS‐CoV‐2 peptides are different in individuals recovered from mild COVID‐19 at 3, 6 and 9 months after recovery within different age groups.

## MATERIAL AND METHODS

### Study participants

A cohort of volunteers in a German SARS‐CoV‐2 high incidence area were recruited for sampling to determine the seropositivity prevalence in the population; the results of which were described elsewhere [20] (German Clinical Trials Register https://www.drks.de ID: DRKS00021306). A follow‐up study to re‐visit these individuals was approved and individuals were sampled 3 months and 6 months after the initial visit (German Clinical Trials Register, https://www.drks.de, ID: DRKS00023113). The study was approved by the Ethics Committee of the Medical Faculty of University Bonn (approval number 085/20). In all visits, plasma was collected and PBMCs isolated through density centrifugation and cryopreserved for downstream analyses. Study participants across all three visits are detailed in Table 1 with reported symptom severity within age groups outlined in Table 2. Participants who were vaccinated or PCR positive for SARS‐CoV‐2 (methods described elsewhere, [20]), at any time‐point, were not included within this study. SARS‐CoV‐2 naïve individuals were sampled from individuals within the first visit group.

**TABLE 1:** Study participants. V1 < 3‐month recovery due to the proximity to the arrival of the SARS‐CoV‐2 virus in Europe in January 2020 ‐ WHO situation reports [42], V2 (6–9‐month recovery), V3 (9–12 months recovery). No individuals were vaccinated at the time of study participation

PBMC collection visit	Number of participants	IgG positive	IgG positive			
			<20‐year old	20–39‐year old	40–59‐year old	>60‐year old
V1	730	154	14	45	65	30
V1 + V2	236	62	7	11	35	9
V1 (+/− V2) + V3	161	43	5	7	25	6

**TABLE 2:** Study participant COVID‐19 symptoms within age groups. No individuals were hospitalized due to COVID‐19. Some individuals reported multiple symptoms

Age groups (years)	Number of participants	Asymptomatic		Anosmia		Cough		Fever	
		Individuals	% of total age group	Individuals	% of total age group	Individuals	% of total age group	Individuals	% of total age group
<20	14	5	35.7%	1	7.14%	7	50%	1	7.14%
20–39	45	6	13.3%	15	33.3%	22	48.9%	13	28.9%
40–59	65	7	10.8%	20	30.8%	35	53.8%	12	18.4%
>60	30	7	23.3%	9	30%	19	63.3%	7	23.3%

### Measurement of SARS‐CoV‐2 spike‐specific plasma IgA and IgG

Cryopreserved plasma samples were thawed and each sample was tested using the Euroimmun IgG and IgA Anti‐SARS‐CoV‐2 ELISA kits, as per manufacturer's instructions (Euroimmun). Briefly, plasma samples were thawed and diluted 1:101 in sample buffer and diluted samples were pipetted into test tubes precovered with SARS‐CoV‐2 spike protein and incubated 60 min at 37°C. Samples were washed thrice and subsequently incubated with peroxidase‐conjugated anti‐IgA or anti‐IgG antibody. After the incubation (30 min at 37°C), the chromogenic substrate was added and samples were incubated for 30 min at room temperature. Finally, the reaction was stopped by the addition of the stop solution, and absorbance was measured on EUROIMMUN Analyzer I platform. A sample was considered positive if the ratio between the absorbance of the sample and calibrator was above 1.0 and negative if below 0.4 for both IgA and IgG.

### T‐cell stimulation

Cryopreserved PBMCs were thawed, washed and rested overnight at 37°C and 5% CO_2_ in RPMI 1640 (Gibco) media containing 5% human AB serum (PAN‐Biotech), 2 mM L‐Glutamine, 1% penicillin/streptomycin and 5 U/ml Benzonase Nuclease HC, Purity >99% (Millipore). Next, PBMCs were analysed for viability (7AAD staining solution, Miltenyi Biotec) and absolute count by MACSQuant® Analyzer 16 (Miltenyi Biotec) and approximately 1x10^6^ PBMCs per well were seeded in U‐bottomed 96‐well plates (Corning) in 100 μl cell culture medium containing 5% human AB serum, 2 mM L‐Glutamine and 1% penicillin/streptomycin per well. Cells were then stimulated via SARS‐CoV‐2 T‐Cell Analysis Kit (PBMC), human (Miltenyi Biotec). In short, cells were stimulated with different SARS‐CoV‐2 PepTivator® Peptide Pools (Prot_S, Prot_S1 and Prot_N) at 1 μg/ml and CytoStim™, human (Miltenyi Biotec) was used as a positive control and DMSO at identical concentrations as stimulated wells as negative control. The peptide pools consist of lyophilized peptides, consisting mainly of 15‐mer sequences with 11 amino acids overlapping. Prot_S mainly covers the S2‐region of the spike protein and is referred to as S2 henceforth. The Prot_S peptide pool covers amino acid regions of S2 including 683–707, 741–770, 785–802 and 885–1273 with a small overlap of the S1 region with the amino acid regions 304–338, 421–475 and 492–519. The Prot_S1 peptide pool completely covers the S1 amino acid regions and is referred to as S1, and the Prot_N peptide pool completely covers the N amino acid regions and is referred to as N. After 2 h stimulation, Brefeldin A (Sigma‐Aldrich) was added to the stimulated cells for a final concentration of 25 μg/ml and incubated for an additional 4 h. Intra‐assay Control (IAC) samples were treated identically but stimulated with a mix of human PepTivator CMV pp65, PepTivator EBV Consensus, PepTivator AdV5 Hexon (Miltenyi Biotec) to illicit a robust response. For this, whole blood from a single healthy individual was collected, processed and preserved identically to study participant samples and used to benchmark assay stability amongst assays across the study.

### ICS for analysis via flow cytometry

The stimulated cells were stained with the live/dead marker Viobility™ 405/452 Fixable Dye, then fixed using Inside Fix (Miltenyi Biotec). Cells were then permeabilized for intracellular staining using the Inside Stain Kit (Miltenyi Biotec) and stained with an anti‐human antibody panel from Miltenyi Biotec for surface proteins CD3 (APC), CD4 (VioBright515), CD8 (VioGreen), CD14 (VioBlue), CD20 (VioBlue), CD154 (APC‐Vio770) and intracellular staining for IFNγ (PE), IL‐2 (PE‐Vio 615) and TNFα (PE‐Vio770).

### Flow cytometry and analysis

Two 96‐well plates each containing 11 study participant samples and one IAC were prepared for analysis every day. Samples were transferred to U‐bottomed 96‐well plates and analysed overnight using two MACSQuant® Analyzer 16 equipped with robotic needle arm, MACS® MiniSampler Plus automated 96‐well plate handler and MACSQuant® Buffer Supply Station 20 L. Over 150 plates were analysed in this manner over a span of 6 months.

Resulting MQD data files were exported in FCS 3.1 format and analysed in FlowJo 10.6.2 (FlowJo, LLC). Gates were drawn upon lymphocytes, single cells, CD3^+^ cells and viable cells before downstream analyses. Gates for activation marker positive CD4 T cells were drawn using unstimulated DMSO control for each donor. Donors with total live CD4 T‐cell counts under 15 000 events were discarded.

### Proliferation assay

Cryopreserved PBMCs were thawed, washed and left overnight in RPMI 1640 (Gibco) media containing 5% human AB serum (PAN‐Biotech), 2 mM L‐Glutamine, 1% penicillin/streptomycin and 5 U/ml Benzonase Nuclease HC, Purity >99% (Millipore). The next day, viable cells were counted via trypan blue exclusion and plated in media containing 5% human AB serum (PAN‐Biotech), 2 mM L‐Glutamine and 1% penicillin/streptomycin at 375000 cells per well into 96‐well flat‐bottomed plate (Corning) in triplicate and stimulated at 0.25 μg/ml with Prot_N (N), Prot_S1 (S1), Prot_S (S2) (Miltenyi Biotec), as well as positive control (PHA 0.5 μg/ml) and negative control (DMSO). Plates were incubated for 48 h. Proliferation was detected using a BrdU Cell Proliferation Assay kit (Sigma) as per manufacturer instructions. In short, BrdU labelling solution was added to wells for the last 20 h of incubation, cells were then dried, fixed and incubated for 90 min with anti‐BrdU‐POD (peroxidase) monoclonal antibody. Next, substrate solution (Tetramethylbenzidine) was added and colour change halted after 5 min with 25 μl 1 M H_2_SO_4_. Plates were read in an ELISA reader at 450 nm. Data are shown as stimulation index (SI) as a fold change compared to DMSO control wells.

### Statistical analyses

Data collection and analysis were performed using FlowJo 10 and GraphPad Prism 9.1. Data are shown as mean + 95% confidence intervals. Activation marker positive T cells were calculated by subtracting the unstimulated DMSO control for each donor with negative values being interpreted as zero. In all figures, * represents a p value of <0.05, **p < 0.01, ***p < 0.001 and ****p < 0.0001.

## RESULTS

### Persistence of SARS‐CoV‐2‐reactive adaptive immune responses after recovery from COVID‐19

**FIGURE 1:**
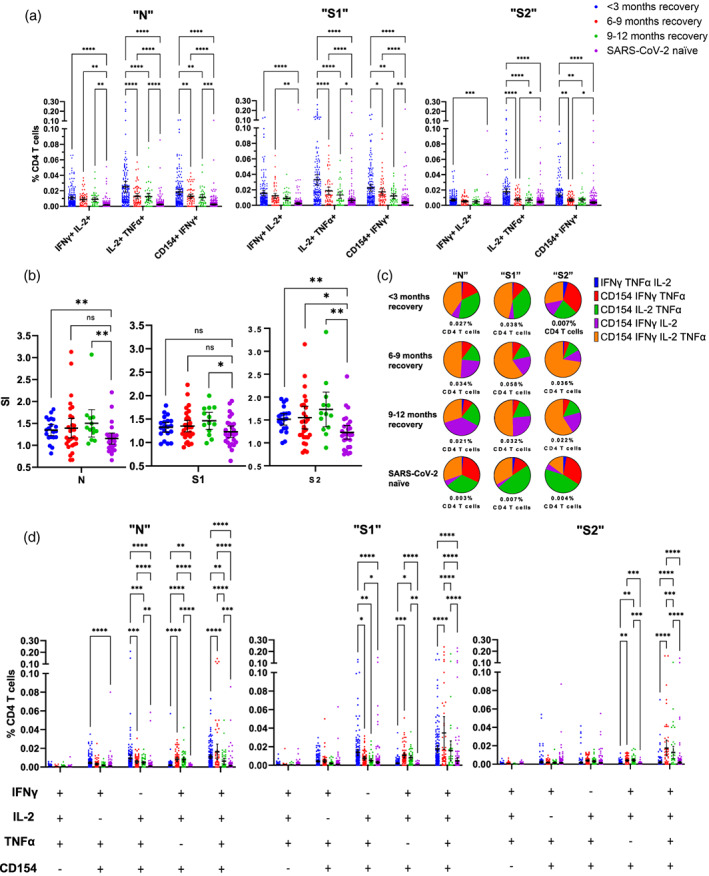
N‐, S1‐ and S2‐induced immune activation over 9 months in individuals recovered from mild COVID‐19. (a) Comparison of dual‐functional activation marker‐positive CD4 T‐cell responses from three time‐points over 9 months from SARS‐CoV‐2‐recovered or naïve individuals is shown after 6‐h incubation with N, S1 or S2 peptide pools. (b) Comparison of SARS‐CoV‐2 N‐, S‐ and S2‐related peptide‐induced proliferative responses from PBMCs taken from SARS‐CoV‐2‐recovered or naïve individuals. Data are shown as fold change compared to DMSO control proliferation (Stimulation Index, SI). Group <3‐month recovery *n* = 20, 6–9‐month recovery *n* = 29, 9–12 months recovery *n* = 30, SARS‐COV‐2 naïve *n* = 27. Data are represented as mean + 95% confidence intervals with differences between groups assessed using Mann–Whitney corrected for multiple comparisons using the Holm‐Šídák method *p < 0.05, **p < 0.01, ***p < 0.001, ****p < 0.0001. (c) Proportional analyses of three‐to‐four‐functional marker‐positive CD4 T‐cell responses over 9 months from SARS‐CoV‐2 recovered or naïve individuals after incubation with N, S1 or S2 peptide pools. Data show mean values as a percentage of all three‐to‐four‐functional CD4 T cells measured which is stated below each pie chart. The frequency of these poly‐functional responses can be seen in D. Group <3‐month recovery *n* = 154, 6–9‐month recovery *n* = 62, 9–12‐month recovery *n* = 43, SARS‐COV‐2 naïve *n* = 327. Data are represented as mean + 95% confidence intervals. Differences between groups were assessed using two‐way ANOVA with Tukey's multiple comparisons test *p < 0.05, **p < 0.01, ***p < 0.001, ****p < 0.0001

Previous studies have demonstrated that the presence of SARS‐CoV‐2‐reactive T cells within an individual is protective against severe COVID‐19 outcome [5] [21]. To quantify SARS‐CoV‐2 reactive CD4 T cells in individuals who recovered from COVID‐19 at least 9 months post recovery, PBMCs were cultured with peptide pools from the Nucleocapsid (N) or Spike (S1) (S2) regions of SARS‐CoV‐2 and CD4 T cells were analysed via flow cytometry for upregulation of Th1‐related cytokines IFNγ, IL‐2 and TNFα as well as expression of CD154 (representative gating shown in Figure S1). Analysis of SARS‐CoV‐2‐reactive CD4 T cells with two upregulated activation markers showed the strongest response at the earliest time‐point, less than 3 months after initial infection which then decreased in magnitude over the next 9 months (Figure 1a). S1 and N, but not S2‐reactive CD4 T cells with two upregulated functional activation markers were still detectable in individuals who recovered from COVID‐19 at least 9 months after recovery when compared to SARS‐CoV‐2 naïve individuals (Figure 1, A, 9–12 months recovery). Analyses of SARS‐CoV‐2‐peptide‐induced increase in proliferation revealed significant proliferative responses at less than 3‐month recovery with N and S2‐peptide pool stimulation (Figure 1b, <3‐month recovery, N *p* = 0.007, S2 *p* = 0.001), at 6‐month recovery with S2 but not N or S1 stimulation (Figure 1b, 6–9‐month recovery, S2 *p* = 0.045) and at 9‐month recovery with all three peptide pools used (Figure 1b, 9–12 months recovery, N *p* = 0.002, S1 *p* = 0.43, S2 *p* = 0.001).

Taken together, these data indicate individuals who recovered from COVID‐19 retain CD4 T cells capable of responding to SARS‐CoV‐2 peptides at least 9 months after recovery towards both the spike and nucleocapsid of SARS‐CoV‐2.

### Polyfunctional SARS‐CoV‐2‐reactive CD4 T‐cell responses over time

Given that polyfunctional T‐cell responses are closely linked to viral control in other viral infections [22, 23], polyfunctional SARS‐CoV‐2‐reactive CD4 T cells were visualized proportionally (Figure 1c) as well as analysed quantitatively (Figure 1d) at 3, 6 and 9 months after recovery from COVID‐19.

From PBMCs collected 6 months after recovery from COVID‐19, percentages of four‐functional CD4 T cells which were induced by N, S1 and S2‐peptides to upregulate IFNγ, IL‐2, TNFα and CD154 were significantly increased compared to SARS‐CoV‐2 naïve‐CD4 T‐cell responses (Figure 1d, n *p* < 0.0001, S1 *p* < 0.0001, S2 *p* < 0.0001), as well as compared to representative responses from the 3 months recovered individuals (Figure 1d, n *p* < 0.0001, S1 *p* < 0.0001, S2 *p* < 0.0001). This trend of increasing poly‐functional CD4 T cells after 6 months post recovery can also be visualized in Figure 1c, with total polyfunctional CD4 T cells increasing from 3 months to 6 months post recovery with all three peptide pools (Figure 1c “N” 0.027%–0.034%, “S1” 0.038%–0.058%, “S2” 0.007%–0.036%), as well as CD154^+^ IFNγ^+^ IL‐2^+^ TNFα^+^ CD4 T cells (Figure 1c “N” “S1” “S2”‐orange segment <3‐month recovery, 6–9‐month recovery).

Analyses of three‐functional CD4 T‐cell responses over time revealed N and S1 but not S2‐targeted‐peptide pools induced significant upregulation of IL‐2, TNFα and CD154 but not IFNγ in CD4 T‐cell responses, compared to SARS‐CoV‐2 naïve individuals; both N‐ and S1‐induced responses were significantly increased at less than 3‐month recovery (Figure 1d, n *p* < 0.0001, S1 *p* < 0.0001), which then significantly decrease at 6 months (Figure 1d, 6–9‐month recovery, N *p* = 0.007, S1 *p* = 0.015) and 9 months (Figure 1d, 9–12 months recovery, N *p* = 0.001, S1 *p* = 0.004) post recovery, but nevertheless remain increased at all time‐points over responses from SARS‐CoV‐2 naïve. In contrast, S2‐induced three‐functional CD4 T‐cell responses involving IFNγ+ IL‐2+ CD154+ CD4 T cells but not TNFα were similar to N‐ and S1‐induced responses and remained significantly increased compared to SARS‐CoV‐2 naïve responses at 6 (Figure 1d, 6–9‐month recovery, N *p* < 0.0001, S1 *p* < 0.0001, S2 *p* = 0.0002) and 9 months (Figure 1d, 9–12 months recovery, N *p* < 0.0001, S1 *p* = 0.0034, S2 *p* = 0.0007) after recovery. Overall, three‐functional CD4 T‐cell responses (IFNγ+ IL‐2+ CD154+ TNFα‐ CD4 T cells) remained elevated and significantly increased at 6 months post recovery when compared to SARS‐CoV‐2 naïve (Figure 1d, 6–9‐month recovery, N *p* < 0.0001, S1 *p* = 0.0006, S2 *p* = 0.0047). The increase in the proportion of IFNγ+ IL‐2+ CD154+ CD4 T cells over time in COVID‐19‐recovered individuals after stimulation with N and S1 peptide pools is represented in Figure 1c (“N” “S1”‐ purple segment).

Collectively, these data indicate that after 3 months of recovery, less polyfunctional Th1‐directed responses to SARS‐CoV‐2 peptides decrease over time but more polyfunctional CD4 T‐cell responses to SARS‐CoV‐2 involving IFNγ, IL‐2 and CD154 and with or without TNFα continue to increase after more than 3 months of recovery from COVID‐19.

### Association of increased age with SARS‐CoV‐2‐reactive CD4 T cells in individuals recovered from COVID‐19

**FIGURE 2:**
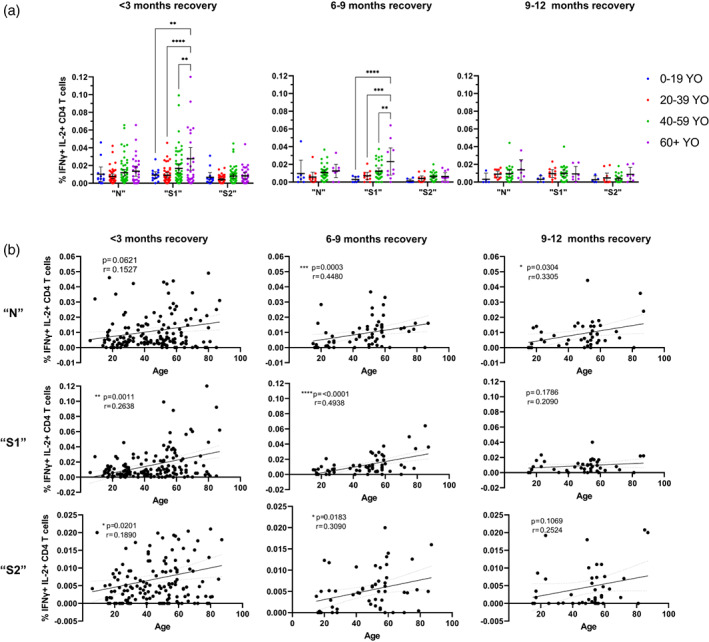
SARS‐CoV‐2‐peptide‐induced challenge responses involving dual‐responding IFNγ+ IL‐2+ CD4 T cells are persistently elevated within older individuals recovered from mild COVID‐19 for at least 9 months post recovery. (a) Comparison of the % of IFNγ+ IL‐2+ positive CD4 T cells after 6‐h incubation with SARS‐CoV‐2 peptide pools N, S1 or S2 peptide pools from individuals recovered from mild COVID‐19 less than 3 months, 6–9 months and 9–12 months post recovery from mild COVID‐19 (IgG≥1). Differences between groups were assessed using two‐way ANOVA with Tukey's multiple comparison test. Group <3‐month recovery 0–19 YO (years old) *n* = 14, 20–39 YO *n* = 45, 40–59 YO *n* = 63, 60+ YO *n* = 31, 6–9‐month recovery 0–19 YO *n* = 7, 20–39 YO *n* = 11, 40–59 YO *n* = 33, 60+ YO *n* = 10, 9–12 months recovery 0–19 YO *n* = 5, 20–39 YO *n* = 11, 40–59 YO *n* = 24, 60+ YO *n* = 7. (b) Correlation and simple linear regression of % of IFNγ+ IL‐2+ CD4 T cells after SARS‐CoV‐2 peptide N, S1 and S2 challenge against age in individuals recovered from mild COVID‐19 < 3‐month post recovery *n* = 122, 6–9‐month post recovery *n* = 60 and 9–12‐month post recovery *n* = 43. Linear regression shown with 95% confidence intervals and effect of age analysed via two‐tailed nonparametric Spearman correlation

**FIGURE 3:**
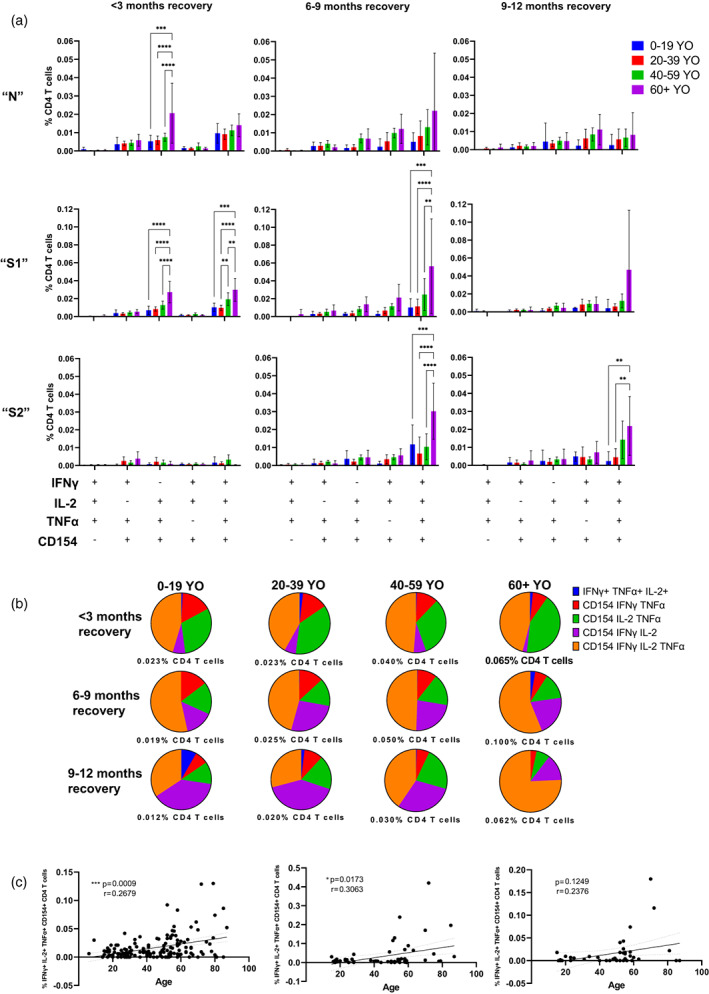
SARS‐CoV‐2‐peptide‐induced challenge responses involving highly poly‐functional CD4 T cells with a Th1 phenotype are persistently elevated within older individuals recovered from mild COVID‐19 for at least 9 months post recovery. (a) Comparison of polyfunctional CD4 T cells with 3–4 functional activation markers from individuals of different age groups at under 3, 6–9 and 9–12‐month recovery from mild COVID‐19 after 6‐h incubation with SARS‐CoV‐2 N, S1 or S2 peptide pools. Differences between groups were assessed using two‐way ANOVA with Tukey's multiple comparisons test *p < 0.05, **p < 0.01, ***p < 0.001, ****p < 0.0001. Data are represented as mean + 95% confidence intervals. Proportional analyses of the S1‐induced polyfunctional CD4 T‐cell responses can be seen in B with the mean values represented as a percentage of all three‐to‐four‐functional responses. Group <3‐month recovery 0–19 YO (years old) *n* = 14, 20–39 YO *n* = 45, 40–59 YO *n* = 63, 60+ YO *n* = 31. Group 6–9‐month recovery 0–19 YO *n* = 7, 20–39 YO *n* = 11, 40–59 YO *n* = 32, 60+ YO *n* = 8. Group 9–12 months recovery 0–19 YO *n* = 5, 20–39 YO *n* = 6, 40–59 YO *n* = 24, 60+ YO *n* = 7. (c) Correlation and simple linear regression of % of IFNγ+ IL‐2+ TNFα+ CD154+ CD4 T cells after SARS‐CoV‐2 peptide S1‐challenge against age in individuals recovered from mild COVID‐19 (<3 months post recovery *n* = 122 (left), 6–9 months post recovery *n* = 60 (middle) and 9–12 months post recovery *n* = 43 (right)). Linear regression shown with 95% confidence intervals and effect of age analysed via two‐tailed nonparametric Spearman correlation

There is evidence to suggest that age is associated with an elevated CD4 T‐cell immune response to SARS‐CoV‐2 in adults compared to children [24]; however, the CD4 T‐cell immune response over time to SARS‐CoV‐2 throughout the ageing population who do not develop severe COVID‐19 is less well studied. To investigate the effect of age on the frequency of SARS‐CoV‐2‐reactive CD4 T cells after incubation with SARS‐CoV‐2 peptides, individuals were grouped by age and CD4 T‐cell responses analysed. The most robust SARS‐CoV‐2‐induced CD4 T‐cell responses were observed in recovered individuals older than 60 years with significantly higher amounts of SARS‐CoV‐2‐reactive CD4 T cells at 3‐, 6‐ and 9‐month recovery (Figure 2a, 3a). Notably, age was positively associated with significant increases in IFNγ+ IL‐2+ CD4 T cells at under 3 and 6 months post recovery upon challenge with S1 peptide against representative responses from all younger age groups (Figure 2a). More poly‐functional age‐specific increases in SARS‐CoV‐2‐reactive CD4 T cells are seen in Figure 3a with individuals over the age of 60 having elevated responses above those of all other younger age groups at under 3‐month recovery (Figure 3a, <3‐month recovery, N and S1), 6‐month recovery (Figure 3a, 6–9‐month recovery, S1 and S2) and at 9 months post recovery with individuals over the age of 60 having elevated S2 responses over the younger age groups under 40 years of age (Figure 3a, 9–12 months recovery, S2).

Whilst increasing age groups are positively associated with the magnitude of particular polyfunctional CD4 T‐cell responses to SARS‐CoV‐2 peptides, analyses of poly‐functional CD4 T‐cell response profiles can indicate the quality of CD4 T‐cell response, as well as the evolution over time. Analyses of the mean three‐to‐four‐functional S1‐response‐profiles of all age groups are seen over time in Figure 3b. Poly‐functional CD4 T‐cell response profiles to S1‐peptide pools did remain visually comparable between all age groups at 3 and 6 months of recovery and are seen in Figure 3b (<3‐month recovery, 6–9‐month recovery); however, older groups progressively recruited increased percentages of total three‐to‐four‐functional CD4 T cells at every time‐point (Figure 3b <3‐month recovery, 6–9‐month recovery and 9–12‐month recovery‐ % CD4 T cells). The evolution of S1‐reactive polyfunctional CD4 T‐cell profile at 3‐, 6‐ and 9‐month recovery was comparable for age groups under 60 years of age with a general increase over time in the proportion of responding three‐functional CD154^+^ IFNγ^+^ IL‐2^+^ CD4 T cells (Figure 3b 0–19 YO, 20–39 YO, 40–59 YO – purple segment); whereas, in individuals over the age of 60, more poly‐functional CD154^+^ IFNγ^+^ IL‐2^+^ TNFα^+^ CD4 T cells can be seen increasing over time (Figure 3b 60+ YO – orange segment) indicating that individuals of increased age can generate a more polyfunctional SARS‐CoV‐2 response profile.

To further analyse this association of increased age and increased SARS‐CoV‐2‐peptide‐induced CD4 T‐cell responses, correlational analyses comparing age with CD4 T‐cell responses were performed. Indeed 6 months after recovery, the strongest IFNγ+ IL‐2+ CD4 T‐cell responses were detected, which correlated positively with age in response to N (Figure 2b, n, *p* = 0.0003, *r* = 0.45), S1 (Figure 2b, S1, *p* < 0.0001, *r* = 0.49) and S2 peptide pool (Figure 2b, S2, *p* = 0.0183, *r* = 0.31). A similar pattern was detected for the more poly‐functional IFNγ+ IL‐2+ TNFα+ CD154+ CD4 T‐cell response in the presence of S1 peptide pool (Figure 3c, S1). Correlational analyses only 3 months post recovery from COVID‐19 showed only weak correlations upon S1‐peptide challenge in IFNγ+ IL‐2+ CD4 T cells (Figure 2b, <3‐month recovery, *p* = 0.0011, *r* = 0.26) and in IFNγ+ IL‐2+ TNFα+ CD154+ CD4 T cells (Figure 2b, <3‐month recovery, *p* = 0.0009, *r* = 0.27).

Taken together, the detectable increase in adaptive immune responses against SARS‐CoV‐2 peptides within older individuals compared to younger age groups, particularly those under the age of 40, indicates that age may be associated with the development of increased adaptive Th1‐directed immune responses against SARS‐CoV‐2‐infection even when COVID‐19 symptoms are only mild or asymptomatic.

### Increased Th1‐adaptive immune response to SARS‐CoV‐2‐peptide challenge in individuals over 40 years of age

**FIGURE 4:**
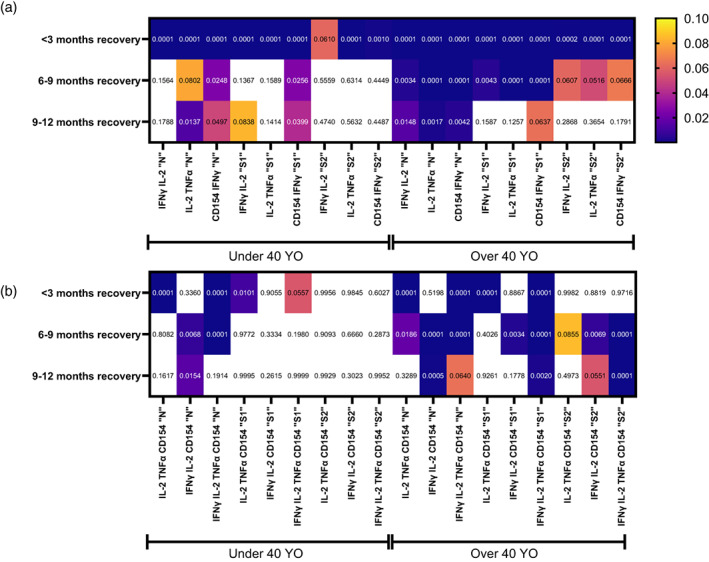
SARS‐CoV‐2‐peptide challenge induced immune activation over 9 months post recovery from mild COVID‐19 indicates persistently increased Th1‐directed peptide challenge responses within individuals over 40 years of age. Heat‐map of p‐values of (a) dual‐activation marker‐positive CD4 T cells and with (b) poly‐functional three‐to‐four activation marker‐positive CD4 T cells from individuals either under or over the age of 40 at under 3, 6–9 and 9–12‐month recovery from mild COVID‐19 after 6‐h incubation with SARS‐CoV‐2 N, S1 or S2‐related peptide pools. Positive responses were compared against age‐matched SARS‐CoV‐2 naïve responses using two‐way ANOVA with Tukey's multiple comparison test. Legend shows *p*‐value scale and exact p‐values are shown within individual boxes. White values denote *p*‐values above 0.1. Group Under 40 YO <3‐month recovery *n* = 59, 6–9‐month recovery *n* = 18, 9–12 months recovery *n* = 12, SARS‐CoV‐2 naïve *n* = 121. Group Over 40 YO <3‐month recovery *n* = 93, 6–9‐month recovery *n* = 41, 9–12 months recovery *n* = 31, SARS‐CoV‐2 naïve *n* = 206

To further investigate whether Th1‐directed increases in SARS‐CoV‐2‐challenge responses can be detected at younger ages and to further dissect differences seen thus far, individuals were grouped and multiple comparison analyses were performed on those above and below 40 years of age compared to age‐matched SARS‐CoV‐2 naive. Almost all analyses involving dual‐functional activation markers at under 3‐month recovery were significantly increased within the under 40‐year olds, with two readouts remaining elevated in this group over SARS‐CoV‐2 naïve at 6 months post recovery (Figure 4a, 6–9‐month recovery, N, CD154+ IFNγ+ *p* = 0.0248 and S1 CD154+ IFNγ+ *p* = 0.0256) and three readouts at 9 months (Figure 4a, 9–12 months recovery, N, IL‐2+ TNFα+ *p* = 0.0137, CD154+ IFNγ+ and S1 CD154+ IFNγ+). Within the over 40‐year‐old group, all dual‐functional activation marker analyses were statistically increased at under 3‐month recovery (Figure 4, A, <3‐month recovery), all N‐ and S1‐induced responses at 6 months post recovery (Figure 4a, N and S1, 6–9‐month recovery) and all N‐induced responses at 9 months post recovery (Figure 4a, n, 9–12 months recovery). These data indicate continued Th1‐directed adaptive immune capabilities against SARS‐CoV‐2 within both these age groups for at least 9 months post recovery from mild COVID‐19.

The highly polyfunctional Th1‐directed CD4 T‐cell responses with three‐to‐four upregulated functional markers after N‐challenge yielded similar patterns in both the under and over 40‐year old with detectable responses at 3, 6 and 9 months post recovery (Figure 4b). However, S1‐ or S2‐induced highly polyfunctional CD4 T‐cell Th1 responses within the under 40‐year‐old group were not detectable when compared to age‐matched naïve responses at any time‐point post recovery (Figure 4b, Under 40 YO, S1 and S2); this is in direct contrast with S‐induced poly‐functional responses from the over 40‐year‐old group with significantly increased amounts of SARS‐CoV‐2‐reactive poly‐functional CD4 T cells at 3, 6 and 9 months post recovery (Figure 4b, Over 40 YO, S1 and S2).

Collectively, these data indicate that SARS‐CoV‐2‐induced Th1‐directed adaptive immune responses to peptides generated from the spike and nucleocapsid continue to be present in individuals both under and over 40 years of age and remain detectable for at least 9 months post recovery. However, Th1‐directed SARS‐CoV‐2‐reactive CD4 T‐cell challenge responses were more readily detected in aged individuals, especially with regard to highly‐polyfunctional CD4 T‐cell responses to peptides generated from the spike of the SARS‐CoV‐2 virus.

## DISCUSSION

Our data indicate that increased age is associated with a more robust CD4 T‐cell response to SARS‐CoV‐2‐peptides with a Th1 phenotype in individuals with comparably mild COVID‐19 symptoms and recovery time. Individuals over the age of 60 had significantly more SARS‐CoV‐2‐reactive polyfunctional CD4 T cells with a Th1 phenotype than those of younger age groups which persisted for at least 9 months after recovery when compared to those under the age of 40.

Age has been associated as one of the largest risk factors for adverse COVID‐19 outcomes [11, 12], whilst adaptive immune responses involving SARS‐CoV‐2‐specific T cells in convalescent individuals have been associated with milder COVID‐19‐symptoms [5, 6, 7]. Within this study, we show that in individuals who do not develop severe COVID‐19, age is associated with a more robust polyfunctional Th1‐CD4 T‐cell response to SARS‐CoV‐2 peptides, indicating a protective role for SARS‐CoV‐2‐directed Th1‐CD4 T‐cell responses with increasing age. A Th1‐mediated response, as investigated here, has been regarded as a more protective phenotype when related to SARS‐CoV‐2 immunity [10]; however, it is also related to the general immunopathogenesis of more severe COVID‐19 [25]. The involvement of Th1 immune responses, therefore, seems to be beneficial, however only when SARS‐CoV‐2‐specific. It has also been reported that SARS‐CoV‐2 challenged T‐cell responses from individuals recovered from mild COVID‐19 lean towards a Th2‐phenotype [26], which is also the case with children who similarly tend towards a Th2 phenotype, which may explain the generally milder COVID‐19 symptoms seen within the young [27]. Investigations into a Th2 phenotype were beyond the scope of this project and future investigations into age‐specific differences in those recovered from mild COVID‐19 should include Th2 phenotypic analyses.

Lower symptomatic infection and more beneficial outcome of SARS‐CoV‐2 infection have been associated with a robust adaptive immune response to viral antigen and a growing body of evidence indicates that a successful recovery from SARS‐CoV‐2‐infection relies upon significant T‐cell involvement [5, 7] involving high avidity SARS‐CoV‐2‐specific T cells [28]. In this study, we show that in individuals that recovered from mild COVID‐19, SARS‐CoV‐2‐reactive CD4 T cells have persistent and strong immune responses. The presence of SARS‐CoV‐2‐reactive CD4 T cells is an indication of protective immunity from re‐infection or severe course of infection [29, 30]. Indeed, it has been demonstrated for other related respiratory viruses such as SARS‐CoV‐1 [31] that induced memory T cells which were detectable 17 years after infection [32]. As with data shown here, others have also shown SARS‐CoV‐2 antigen‐induced T‐cell responses in convalescent individuals which persist for at least 6 months and then begin to wane [33, 34, 35, 36].

Polyfunctional T cells have been strongly linked with an increased ability to combat viral infections [22, 23] and early protection, even before antibody production [21]. The highly polyfunctional SARS‐CoV‐2‐reactive CD4 T cells upregulating functional activation markers IFNγ, IL‐2 and CD154 (which were also either TNFα positive or negative) peaked after around 6‐month recovery. This increase was also accompanied with a subsequent steady decrease in polyfunctional CD4 T cells which were not upregulating IFNγ. This shift towards a larger percentage of polyfunctional CD4 T cells upregulating IFNγ and TNFα could be attributed to differentiation towards a Th‐1 central memory phenotype, which has been shown to be increasing until at least 6 months post symptom onset [33] and is consistent with polyfunctional CD4 T‐cell responses in individuals infected and challenged with SARS‐CoV‐1‐peptides [37].

The effect of ageing on the immune system can be characterized by a higher initial pro‐inflammatory status alongside a progressive inability of the immune system to mount efficient responses [38]. A potential explanation for this is an association with age and a progressive decrease in naïve T‐cell populations [18], which has also been associated with more severe COVID‐19 symptoms [5]. It is possible that a reduction in naïve T cells with age leads to a delayed adaptive immune response to the novel SARS‐CoV‐2 antigens, which, even in those individuals who develop mild symptoms, have a dampened response to the virus, leading to an increase in viral load, a more drawn‐out recovery, and a more robust CD4 T‐cell response to SARS‐CoV‐2 peptides upon challenge.

The increase in SARS‐CoV‐2‐reactive polyfunctional CD4 T cells at 6 months post recovery was not accompanied with an increase in mono‐functional (data not shown) or dual‐functional activation marker‐positive SARS‐CoV‐2‐reactive CD4 T cells which were equal to or significantly decreased at 6‐month recovery compared to at under 3‐month recovery, yet still significantly increased over SARS‐CoV‐2‐naïve responses. Corroborating publications towards a robust CD4 T‐cell response at around 6‐month recovery from COVID‐19 also indicate the involvement of polyfunctional T cells involving IL‐2, IFNγ and TNFα [29, 30]. Additionally, decreases in mono‐functional T‐cell responses to S‐peptides have been reported previously at 5 months after diagnosis of SARS‐CoV‐2‐infection in health‐care workers; however, polyfunctional T‐cell responses were not analysed [39]. Limitations within this study include the use of PBMCs to investigate SARS‐CoV‐2‐reactive CD4 T cells, excluding resident memory T cells, which form an integral part of the immune homeostasis and cannot be sampled from venous blood [40]. Further studies should include longevity and age‐associated SARS‐CoV‐2‐reactive tissue‐specific T‐cell analyses.

Immune responses in SARS‐CoV‐2 naïve individuals in particular towards S but not towards N, have been reported, with around 40–60% of unexposed donors responding to SARS‐CoV‐2 peptides [41]. As it was not possible to exclude which of the SARS‐CoV‐2‐naïve donors in this study had cross‐reactive T cells, it is likely that analyses comparing S1 and S2‐reactive CD4 T‐cell responses in SARS‐CoV‐2‐exposed, against SARS‐CoV‐2‐naïve, are less significant than if those cross‐reactive naïve individuals were excluded, as would be ideal.

Taken together, our findings indicate that in individuals who recovered with comparably mild COVID‐19 symptoms, SARS‐CoV‐2‐reactive CD4 T cells are still detectable at least 9 months after SARS‐CoV‐2 recovery. We show that age is associated with significantly more robust Th1‐directed CD4 T‐cell responses, with higher amounts of SARS‐CoV‐2‐reactive polyfunctional CD4 T cells in individuals over the age of 60 compared to those under the age or 40. However, we show that some SARS‐CoV‐2‐reactive CD4 T cells with three to four Th1‐directed positive functional activation markers remain comparable or increased at over 9‐month recovery when compared to at under 3‐month recovery, including in individuals under 40 years of age. The reduced CD4 T‐cell Th1 responses to SARS‐CoV‐2 peptide‐challenge in individuals recovered from mild COVID‐19 under the age of 40 raises questions regarding the long‐term protective immunity that natural infection imbues in the young.

## CONFLICT OF INTEREST

The authors declare no competing interests.

## AUTHOR CONTRIBUTIONS

RGN, PZ, MS and BK were responsible for experimental design. RGN performed experiments with the help of LK, CS, JB and EB. RGN analysed the data and wrote the manuscript which was read and revised by all other authors. LS furthermore contributed to the project through the context of her master thesis. PZ and BK were responsible for project logistics, mechanical upkeep and technical advice. HS supervised the project and revised the manuscript.

## Supporting information


Figure S1

## Data Availability

The data that support the findings of this study are available from the corresponding author upon reasonable request.
