# Supplementary figures and images for: The effect of age on the magnitude and longevity of Th1‐directed CD4 T‐cell responses to SARS‐CoV‐2
Source: Immunology. 2022 Apr 22;166(3):327–40. doi: 10.1111/imm.13475 (PMC9111694; doi:10.1111/imm.13475)

## Slide 1
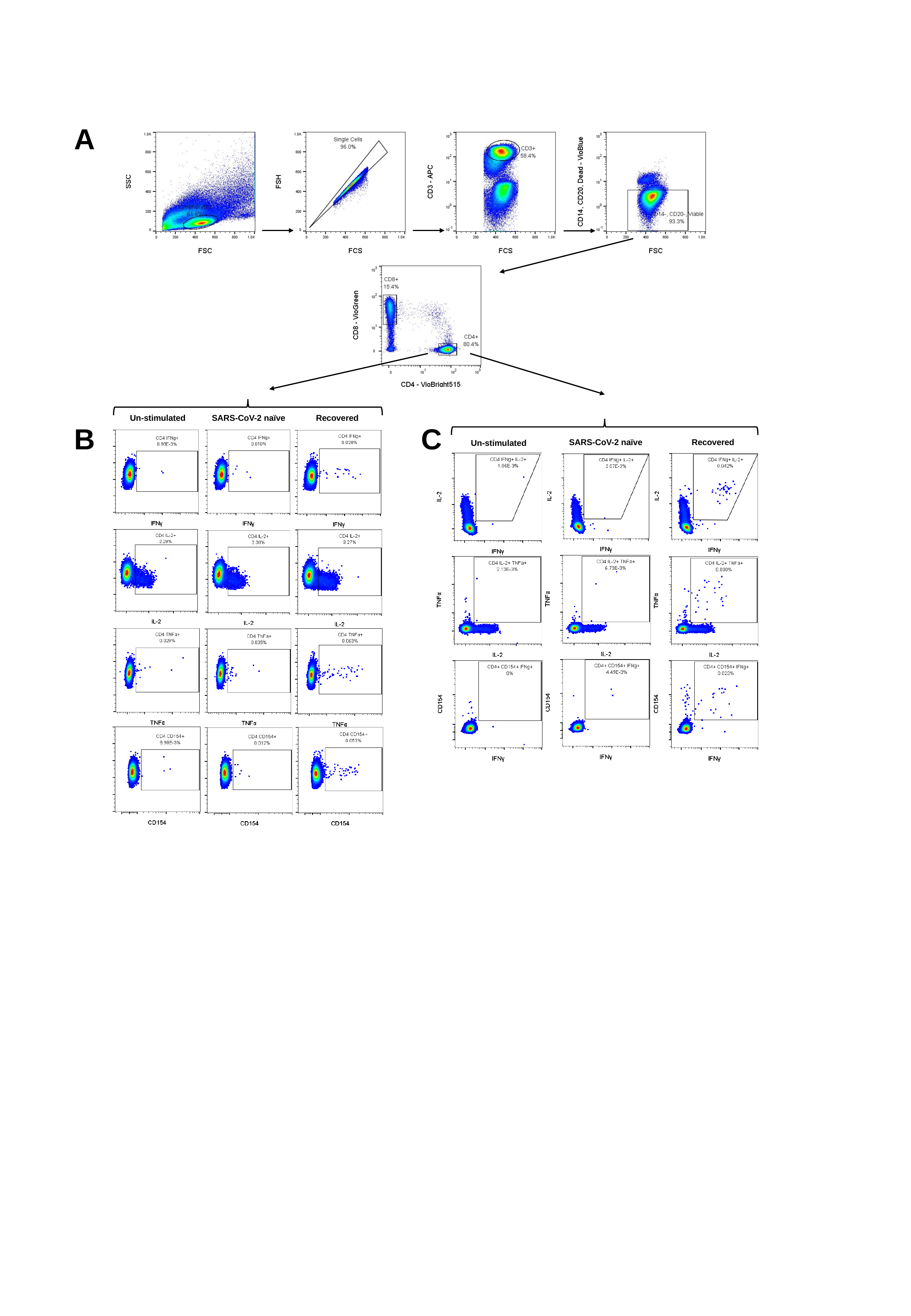

A
SARS-CoV-2 naïve
Recovered
Un-stimulated
B
C
SARS-CoV-2 naïve
Recovered
Un-stimulated

Supplement: Supplementary file 1 — Figure S1 [file IMM-166-327-s001.pptx]
